# Construct validity, test–retest reliability and internal consistency of the Thai version of the disabilities of the arm, shoulder and hand questionnaire (DASH-TH) in patients with carpal tunnel syndrome

**DOI:** 10.1186/s13104-018-3318-5

**Published:** 2018-03-27

**Authors:** Montana Buntragulpoontawee, Suphatha Phutrit, Siam Tongprasert, Tinakon Wongpakaran, Jeeranan Khunachiva

**Affiliations:** 1 Department of Rehabilitation Medicine, Faculty of Medicine, Chiang Mai University, Chiang Mai, 50200 Thailand; 2 Department of Psychiatry, Faculty of Medicine, Chiang Mai University, Chiang Mai, Thailand

**Keywords:** DASH-TH, Reliability, Validity, Internal consistency, Carpal tunnel syndrome, Factor structure

## Abstract

**Objective:**

This study evaluated additional psychometric properties of the Thai version of the disabilities of the arm, shoulder and hand questionnaire (DASH-TH) which included, test–retest reliability, construct validity, internal consistency of in patients with carpal tunnel syndrome. As for determining construct validity, the Thai EuroQOL questionnaire (EQ-5D-5L) was also administered in order to examine convergent and divergent validity.

**Results:**

Fifty patients completed both questionnaires. The DASH-TH showed excellent test–retest reliability (intraclass correlation coefficient = 0.811) and internal consistency (Cronbach’s alpha = 0.911). The exploratory factor analysis yielded a six-factor solution while the confirmatory factor analysis denoted that the hypothesized model adequately fit the data with a comparative fit index of 0.967 and a Tucker–Lewis index of 0.964. The related subscales between the DASH-TH and the Thai EQ-5D-5L were significantly correlated, indicating the DASH-TH’s convergent and discriminant validity. The DASH-TH demonstrated good reliability, internal consistency construct validity, and multidimensionality, in assessing the upper extremity function in carpal tunnel syndrome patients.

**Electronic supplementary material:**

The online version of this article (10.1186/s13104-018-3318-5) contains supplementary material, which is available to authorized users.

## Introduction

Carpal tunnel syndrome (CTS) or median neuropathy at the wrist commonly presents with numbness, dysesthesia and weakness of the hand(s), resulting in significantly limited functioning. It has been described as the most commonly diagnosed entrapment neuropathy in clinical practice, with a clinically and electrophysiologically confirmed CTS prevalence of 2.7% [1]. To assess the functional impact of CTS in the clinic, many clinical questionnaires have been developed, for example, a self-administered questionnaire by Levine (1993) and the Boston questionnaire [2, 3]. In 2008, Kumnerdee and Uphatham translated the Boston questionnaire into Thai and tested only its internal consistency [4]. In addition to the CTS-specific outcome measurement tools, the disabilities of the arm, shoulder and hand (DASH) outcome measure demonstrated compatibility with the Boston questionnaire and was previously translated into Thai (DASH-TH) with good content validity and high internal consistency. The DASH’s primary clinical advantage is that it serves as a single, reliable instrument that can assess any or all joints in the upper extremities, rather than requiring a specific instrument for each joint or diagnosis. The DASH-TH is clearly preferable; however, the psychometric properties of the Thai version remain unexplored [5, 6]. This study evaluated the construct validity, internal consistency, and test–retest reliability of the DASH-TH in CTS patients.

## Main text

### Methods

**Fig. 1:**
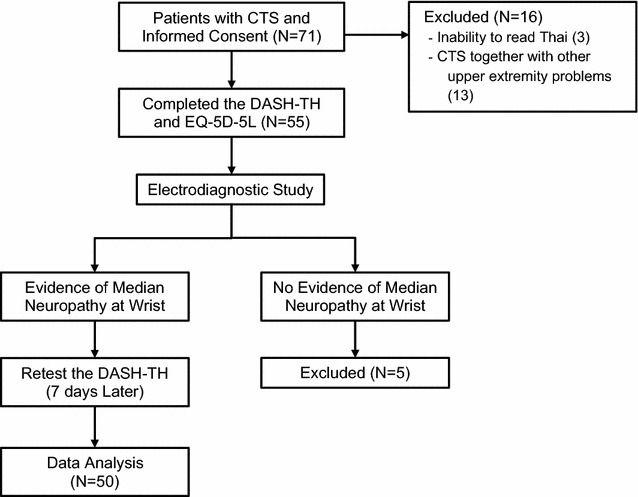
Flow diagram demonstrating enrollment, electrodiagnostic procedure and data collection

CTS patients between 18 and 80 years of age from the general rehabilitation out-patient clinic at Maharaj Nakorn Chiang Mai Hospital were considered for inclusion in this study. All participants completed at least elementary school grade six and could read and understand Thai. Patients were excluded if they had other upper extremity problems, TBI, mood disorder, or no electrodiagnostic evidence of CTS. Study information was provided by the study team and informed consent was obtained. Demographic information was collected from the eligible patients, who then answered the DASH-TH and the Thai EQ-5D-5L. An electrodiagnostic (EDx) appointment was made 1 week later, but no treatment was provided at that time. Once the CTS diagnosis was confirmed by EDx, the patient remained in the study and completed the DASH-TH a second time (Fig. 1).

#### DASH questionnaire

The DASH questionnaire consists of 30 self-reported disability and symptom questions. Since no a priori model of the DASH [7] exists, we hypothesized, based on item characteristics, that the DASH included five subscales, consisting of common activities (20 items, items 1–12 and 16–23), self-care activities (3 items, items 13–15), pain symptoms (2 items, items 24–25), other symptoms such as numbness, joint stiffness, weakness, and sleep problems (4 items, items 26–29) and psychological effects (1 item, item 30). Examples of these DASH items included: “turn a key”, “change a light bulb overhead”, and “use a knife to cut food”. The scores ranged from 0 to 100 with higher scores indicating greater disability [6].

#### EQ-5D-5L

The EQ-5D-5L is a common, generic instrument for evaluating health outcomes. It has been translated into Thai with all psychometric properties being evaluated [8–10]. The score was calculated using a Thai valuation set of the EQ-5D-5L [8], which was divided into two sections. The first section contained five dimensions: mobility, usual activities, self-care activities, pain symptoms and anxiety/depression. The index values ranged from − 1 to 1, with higher values indicating better health. The second part evaluated health conditions using a direct measurement, the visual analogue scale (VAS), ranging from the worst health condition (0) to the best possible health condition (100).

Data analysis was performed using IBM SPSS 22 for Windows (IBM Corporation, Armonk, NY, USA). Descriptive variables are presented as percentages, means, standard deviations (SD) and ranges. The Shapiro–Wilk test was used to assess data distribution.

#### Construct validity

Exploratory factor analysis (EFA) was used to investigate construct validity by factor structure. Since the response was a categorical variable, polychoric correlation with matrix with weighted least square parameter estimation was used Mplus 7.4 software (Muthén & Muthén, Los Angeles, CA, USA). Due to the small sample size, 40 samples consisting of CTS and common upper extremity musculoskeletal disorders from our previous study were added to have sufficient data for the factorial analysis [6].

Confirmatory factor analysis (CFA) was performed for a hypothesized five-model structure to compare the extent of fit indices with various models yielded by EFA. We used the following fit indices: a comparative fit index (CFI) of ≥ 0.95, a non-normed fit index (NFI) or a Tucker–Lewis index (TLI) of ≥ 0.9, and a root-mean-square error of approximation (RMSEA) of ≤ 0.6. Values as high as 0.08 [11–13], a weighted root mean square residual (WRMR) < 0.9 [14] and a χ^2^/*df* result of < 3 indicated a reasonable fit [15].

Construct validity was also examined by convergent and divergent validity assessment, both measuring Spearman’s rank correlation coefficient (r) of related and different dimensions between the EQ-5D and DASH subscales. Two hypotheses were examined: (1) The same dimensions of the DASH-TH and EQ-5D-5L questionnaires are associated, including normal activities, self-care activities and pain symptoms. (2) Different dimensions are unassociated or weakly associated, including self-care activities on the DASH-TH and mobility on the Thai EQ-5D-5L.

The correlation strength was determined as follows: absent or weak (r < 0.25), moderate (0.25 < r < 0.50), good (0.50 < r < 0.75), and excellent (r > 0.75) [16].

#### Internal consistency

Internal consistency of the DASH-TH was assessed using Cronbach’s alpha coefficient with the acceptable value being 0.70–0.95 [17].

#### Test–retest reliability

Test–retest reliability of the DASH-TH was assessed after 7 days by an intraclass correlation coefficient (ICC). The coefficient can range from 0 to 1, and a coefficient > 0.7 indicates good reliability [17].

### Results

Seventy-one patients with clinically suspected CTS participated in the study. Twenty-one patients were excluded, with 50 patients (male 8, female 42) remaining (Fig. 1). Of the total 81 CTS hands, 34 hands (42%) were severely affected by grading the carpal tunnel syndrome severity using the electrodiagnostic reports of Sucher BM (Table 1) [18].

**Table 1:** Demographic characteristics

Characteristics	Mean (SD)
Age (years)	53.92 (12.17) (Min 29, max 74)
Duration of symptoms (months)	7.26 (10.03)

Characteristics	N (%)
Gender	
Male	8 (16)
Female	42 (84)
Affected sides	
Left	10 (20)
Right	9 (18)
Both	31 (62)

Characteristics	N (%)
Severity of CTS	
Mild	23 (28)
Moderate	24 (30)
Severe	34 (42)

*N* number of hands

The range score for the initial DASH-TH was 8.30–67.50 with an average score of 27.96 (SD = 13.90). The re-test DASH-TH score ranged from 7.00 to 64.2 with an average score of 27.38 (SD = 15.21). Both had similar average scores, and no patients had a maximum (ceiling) or minimum (floor) score. The EQ-5D-5L index values ranged from 0.097 to 1, and the average value was 0.575 (SD = 0.167). The EQ-VAS values ranged from 50 to 100, and the average score was 72.10 (SD = 13.48). One patient scored 100, which is the highest possible score on both the EQ-5D-5L and EQ-VAS. All patients completed both questionnaires independently without help.

Despite the relatively small number of subjects, the Kaiser–Meyer–Olkin measure (KMO) coefficient of 0.86, indicated an adequate sample size for conducting the factor analysis.

A factor analytic study was unfit for the one-factor model, while yielding the best model fit with a six-factor solution (Chi square 390.704, df = 270, RMSEA = 0.070 [0.054, 0.085], CFI = 0.993, TLI = 0.989, SRMR = 0.042). Our hypothesized five-factor model had a fair level of fit indices; however, they were less fit than the six-factor model (Table 2). Exploratory factor analysis using eigenvalues > 1 extracted greater than seven factors. Several cross-loading items were loaded on the same factor, e.g. item 1 (open a tight or new jar) and item 22 (interference in social activities); item 8 (garden or do yard work) and item 23 (limited in your work or other regular daily activities). The one-factor model was a poor fit for the sample, while the six-factor solution was the best model for the data (Chi square 390.704, df = 270, RMSEA = 0.070 [0.054, 0.085], CFI = 0.993, TLI = 0.989, SRMR = 0.042).

**Table 2:** Comparing fit statistics among models (N = 90)

Fit indices	Hypothesized-5 factor^a^	Six-factor	Five-factor	Four-factor	Three-factor	Two-factor	One-factor
Chi square	976.995	390.704	518.185	685.468	840.903	1228.741	1266.713
Df	395	270	295	321	348	376	405
RMSEA	0.128 (0.118 0.138)	0.070 (0.054 0.085)	0.092 (0.079 0.105)	0.112 (0.101 0.124)	0.125 (0.115 0.136)	0.159 (0.115 0.169)	0.206 (0.193 0.219)
CFI	0.967	0.993	0.987	0.979	0.972	0.952	0.840
TLI	0.964	0.989	0.981	0.972	0.965	0.944	0.828
SRMR/WRMR	0.968^b^	0.042	0.049	0.059	0.068	0.090	0.152

*Df* degree of freedom, *RMSEA* root-mean-square error of approximation, *CFI* comparative fit index, *SRMR* standardized root-mean-square residual, *TLI* Tucker–Lewis index, *WRMR* weighted root mean square residual

^a^CFA 5 domain: factor 1, 1–16; factor 2, 17–21; factor 3, 22–23; factor 4, 24–28; factor 5, 29–30

^b^This value was calculated by *WRMR*

Confirmatory factor analysis for the hypothesized five-factor model had a fair level of fit indices.

Correlational analysis showed that similar dimensions of both questionnaires were correlated, such as usual activity (r = 0.425), self-care activities (r = 0.532) and pain symptoms (r = 0.351). In contrast, different dimensions were weak to moderately correlated, such as usual activities on the DASH-TH and mobility on the EQ-5D-5L (r = 0.265). These results (Additional file 1) support the hypothesis. The correlation coefficients between the total scores on the DASH-TH and EQ5D-TH and the EQ VAS were 0.572 and 0.672 (all p < 0.01), respectively.

The internal consistency was high (Cronbach’s alpha coefficient = 0.911), and test–retest reliability was excellent, as the ICC was 0.811, 95% Cl 0.668–0.893.

### Discussion

The DASH is a self-reported outcome measure for assessing disability resulting from upper extremity disorders. In 2013, the DASH was translated into Thai (DASH-TH), and its psychometric properties, content validity, and internal consistency were tested. To further evaluate the DASH-TH properties, this study tested the construct validity and test–retest reliability in CTS patients.

Most of the CTS patients were female (84%), which is consistent with studies from Korea (89%), Japan (89%) and England (72%) [5, 19, 20].

Similar to previous studies, the DASH-TH is not confirmed to be a unidimensional model; thus, other DASH versions were created [21–23]. This was expected, as the scale appeared to be designed for addressing multi-domains, i.e. daily (usual) activities, symptoms, social functions, and psychological issues; therefore, it is unlikely that all items measure a single construct.

CFA is sample-dependent; therefore, it tends to have better results with a larger sample size. The sample data may fit better to the hypothesized five-factor model if a larger sample size is adopted instead of the six-factor model.

In addition, internal consistency was re-tested for comparison with the previous DASH-TH study (Cronbach’s alpha = 0.94) [6]. Cronbach’s alpha remained high (0.91), even when limited to CTS patients, implying wide applicability among various upper extremity disorders.

The correlation between DASH-TH and the Thai EQ-5D-5L was -0.572, which was slightly lower than the value obtained by Slobogean et al. [24] in Canada (2010) (r = − 0.75), who studied a larger group of 61 patients with humeral fractures. Nevertheless, a significant moderate-to-good correlation was found between similar dimensions of the DASH-TH and EQ-5D-5L, for example, usual activities, self-care activities and pain, reflecting good convergent validity. Different dimensions were either weakly or not correlated, reflecting good divergent validity.

Test–retest reliability of the DASH-TH was also high (ICC = 0.811), which was consistent with studies in the CTS population by Greenslade 2004 (ICC = 0.9) in England, Imaeda et al. in Japan in 2005 (ICC = 0.82), and Lee et al. in Korea in 2008 (ICC = 0.91) [5, 25, 26]. All test–retest studies used a 1–2-week window, which was the preferred duration as it was too short for the symptoms to change, but long enough that the patient was unlikely to remember their initial answers [17].

### Conclusion

The DASH-TH was shown to be a reliable and valid tool for assessing and following CTS patients’ symptoms, functions, and abilities. It appeared to have factor structure as hypothesized, and the external validations of the scales sufficiently demonstrated the ability to measure upper arm disability. Furthermore, the DASH-TH offers greater clinical utility because it measures comprehensive functioning for any diseases or upper extremity regions, providing practicality in a busy clinical setting where using each disease or region-specific outcome measure would be cumbersome.

## Limitations

One limitation of this study was the small sample size. Although a sample size of 50 patients was sufficient to evaluate convergent and divergent validity, the reliability of each item using item response theory, e.g. Rasch analysis or other IRT models, should be further examined using a larger sample size. Another important aspect of the DASH-TH questionnaire property is its responsiveness, which should be investigated in future studies. Although the DASH can be applied to many sample characteristics, the mixed sample used in the present analysis may impact its factorial validity because there may be differential items functioning between the two samples. Therefore, the factor analysis results should be interpreted cautiously.

## Additional file

**Additional file 1.** Correlation Coefficients (r) Between DASH-TH and Thai EQ-5D-5L. Correlation analysis was performed in order to assess convergent and 370 divergent validity of EQ-5D subscales and DASH subscales. Significant correlations 371 were found between similar dimensions such as, usual activities, self-care, and pain 372 whereas different dimensions had weak to moderate correlation.

## Abbreviations

CTS: carpal tunnel syndrome
DASH: disabilities of the arm, shoulder and hand
ICC: intraclass correlation coefficient
EFA: exploratory factor analysis
CFA: confirmatory factor analysis
EDx: electrodiagnostic
SD: standard deviations
CFI: comparative Fit Index
NFI: non-normed fit index
TLI: Tucker–Lewis index
Df: degree of freedom
RMSEA: root-mean-square error of approximation
WRMR: weighted root mean square residual
