# Supplementary material for: Construct validity, test–retest reliability and internal consistency of the Thai version of the disabilities of the arm, shoulder and hand questionnaire (DASH-TH) in patients with carpal tunnel syndrome
Source: BMC Res Notes. 2018 Mar 27;11:208. doi: 10.1186/s13104-018-3318-5 (PMC5872536; doi:10.1186/s13104-018-3318-5)
Supplement: Supplementary file 1 — Additional file 1. Correlation Coefficients (r) Between DASH-TH and Thai EQ-5D-5L. Correlation analysis was performed in order to assess convergent and 370 divergent validity of EQ-5D subscales and DASH subscales. Significant correlations 371 were found between similar dimensions such as, usual activities, self-care, and pain 372 whereas different dimensions had weak to moderate correlation. [file 13104_2018_3318_MOESM1_ESM.docx]

**Title of data:** Correlation Coefficients (r) Between DASH-TH and Thai EQ-5D-5L

|  | EQ-5D-5L | | | | | |
| --- | --- | --- | --- | --- | --- | --- |
|  | Mobility | Self-Care | Usual Activities | Pain | Anxiety/ Depression |  |
| DASH-TH | 0.312* | 0.572** | 0.437** | 0.235 | 0.344* |  |
| Usual Activities | 0.265 | 0.565* | 0.425** | 0.252 | 0.378** |  |
| Self-Care Activities | 0.198 | 0.532** | 0.301* | 0.215 | 0.151 |  |
| Pain Symptom | 0.136 | 0.321* | 0.306* | 0.351* | 0.096 |  |
| Other Symptoms | 0.283* | 0.269* | 0.168 | 0.117 | 0.170 |  |
| Psychological Effect | 0.174 | 0.182 | 0.240 | 0.172 | 0.153 |  |

**significant at p value <0.01

* significant at p value <0.05
